# The effect of blood cell count on coronary flow in patients with coronary slow flow phenomenon

**DOI:** 10.12669/pjms.305.4935

**Published:** 2014

**Authors:** Korhan Soylu, Okan Gulel, Huriye Yucel, Serkan Yuksel, Gokhan Aksan, Ayşegül İdil Soylu, Sabri Demircan, Özcan Yılmaz, Mahmut Sahin

**Affiliations:** 1 Korhan Soylu, MD, Assistant Professor, Department of Cardiology, Faculty of Medicine, Ondokuz Mayis University, Samsun, Turkey.; 2 Okan Gulel, MD, Associate Professor, Department of Cardiology, Faculty of Medicine, Ondokuz Mayis University, Samsun, Turkey.; 3 Huriye Yucel, MD, Department of Cardiology, Faculty of Medicine, Ondokuz Mayis University, Samsun, Turkey.; 4 Serkan Yuksel, MD, Assistant Professor, Department of Cardiology, Faculty of Medicine, Ondokuz Mayis University, Samsun, Turkey.; 5 Gokhan Aksan, MD, Department of Cardiology, Gazi State Hospital, Samsun, Turkey.; 6 Ayşegül İdil Soylu, MD, Department of Radiology, Samsun Education Research Hospital, Samsun, Turkey.; 7 Sabri Demircan, MD, Associate Professor, Department of Cardiology, Faculty of Medicine, Ondokuz Mayis University, Samsun, Turkey.; 8 Özcan Yılmaz, MD, Professor, Department of Cardiology, Faculty of Medicine, Ondokuz Mayis University, Samsun, Turkey.; 9 Mahmut Sahin, MD, Professor, Department of Cardiology, Faculty of Medicine, Ondokuz Mayis University, Samsun, Turkey.

**Keywords:** Basophil, Coronary slow flow, Eosinophil, Hematocrit, Hemoglobin

## Abstract

***Background and Objective:*** The coronary slow flow phenomenon (CSFP) is a coronary artery disease with a benign course, but its pathological mechanisms are not yet fully understood.The purpose of this controlled study was to investigate the cellular content of blood in patients diagnosed with CSFP and the relationship of this with coronary flow rates.

***Methods:*** Selective coronary angiographies of 3368 patients were analyzed to assess Thrombolysis in Myocardial Infarction (TIMI) frame count (TFC) values. Seventy eight of them had CSFP, and their demographic and laboratory findings were compared with 61 patients with normal coronary flow.

***Results:*** Patients’ demographic characteristics were similar in both groups. Mean corrected TFC (cTFC) values were significantly elevated in CSFP patients (p<0.001). Furthermore, hematocrit and hemoglobin values, and eosinophil and basophil counts of the CSFP patients were significantly elevated compared to the values obtained in the control group (p=0.005, p=0.047, p=0.001 and p=0.002, respectively). The increase observed in hematocrit and eosinophil levels showed significant correlations with increased TFC values (r=0.288 and r=0.217, respectively).

***Conclusion: ***Significant changes have been observed in the cellular composition of blood in patients diagnosed with CSFP as compared to the patients with normal coronary blood flow. The increases in hematocrit levels and in the eosinophil and basophil counts may have direct or indirect effects on the rate of coronary blood flow.

## INTRODUCTION

The coronary slow flow phenomenon (CSFP) is defined as the delay in the progression of the injected contrast material in the distal coronary bed during coronary angiography, in the absence of any luminal narrowing of the vessels.^1^ In the absence of visible narrowing of the epicardial arteries, this syndrome has been referred to as ‘a subtype of syndrome X’. Although generally associated with a benign course, there are some reports linking CSFP with serious cardiac events such as acute myocardial infarction, sudden cardiac death, cardiac dysfunction and fatal arrhythmia.^2^^-^^6^

The hypotheses proposed for the etiology of CSFP mainly concern the increase in the sympathetic activity and microvascular dysfunction. However, it has also been demonstrated that endothelial thickening in small arteries^7^, myocardial fibrosis^8^, disorders of endothelial nitric oxide release^9^, diffuse atherosclerosis^10^ and inflammation^11^ may contribute to the development of this abnormality. Investigations made so far have not taken into account a possible effect of blood cellular composition on coronary flow. The aim of this study was to investigate the possible role of blood cellular composition in the pathophysiology of CSFP.

## METHODS

**Fig.1:**
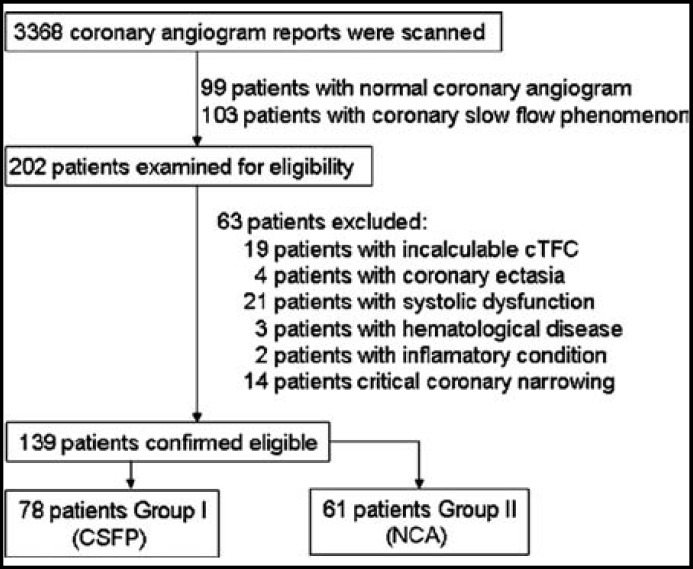
Study flow diagram. CSFP: Coronary slow flow phenomenon

***Study Design:*** This is a retrospective case-control study involving patients who underwent coronary angiography between September 2010 and December 2012 at the Department of Cardiology of the Medical Faculty at Ondokuz Mayis University, Samsun, Turkey. Coronary angiogram reports of 3368 patients were scanned from digital database in our laboratory. One hundred and three coronary angiography reports with SCFP and 99 reports with normal coronary angiograms were selected randomly. Cases with acute coronary syndrome, coronary ectasia or spasm, severe valvular or congenital heart disease, left ventricular systolic dysfunction (EF < 55%), chronic hematological diseases, inflammatory conditions, allergic diseases, autoimmune diseases, parasitic diseases and obstructive coronary lesions were excluded from the intended investigation (Fig.1).

***Coronary Angiography:*** Coronary angiography through the femoral or radial artery using the Seldinger technique was performed on all patients included in the study. The images obtained during the procedure were recorded by means of a digital angiographic system (ACOM.PC; Siemens AG, Germany) at our clinic, which made recordings at a rate of 15 frames/second. These recordings were subsequently examined by two independent cardiologists in order to estimate the coronary blood flow according to the Thrombolysis in Myocardial Infarction frame count (TFC) proposed by Gibson et al.^12^ Since the most frequently standardized filming rate is 30 frames/second, the results obtained were multiplied by two. Finally, TFC values for the left anterior descending artery were divided by 1.7 to obtain the corrected TFC (cTFC).

***Definitions for Coronary Flow and Obstructive Coronary Lesion:*** In accordance with the reports in the literature^12^, threshold values obtained after correction of the cut-off values were 36.2±2.6 frames for the left anterior descending coronary artery, 22.2±4.1 frames for the left circumflex artery, and 20.4±3 frames for the right coronary artery. Patients in the present study who had cTFC values exceeding these thresholds by greater than 2 standard deviations (SD) for the particular vessel were recognized as having CSFP. Patients who had cTFC values not exceeding these thresholds were recognized as having normal coronary flow. Obstructive coronary lesion was defined as that compromising the luminal diameter by 50% or more on coronary angiography. A normal coronary angiogram was defined as one with the absence of any visible angiographic signs of atherosclerosis, thrombosis or spontaneous spasm.

***Blood Sampling:*** Blood samples drawn from the antecubital vein of the patients were collected in EDTA K3 tubes. Hematological parameters were measured using an electronic hematology analyzer (Cell-Dyn Sapphire; Abbott Diagnostic Division, Santa Clara, California, USA).

***Statistical Analyses:*** Statistical analyses were conducted using the Statistical Package for the Social Sciences for Windows 13.0 software (SPSS, Chicago, IL, USA). Descriptive statistics were given as mean, standard deviation, frequency and percentage. The Kolmogorov Smirnov test was used to evaluate whether the continuous variables were normally distributed. For continuous variables the independent samples t-test or Mann Whitney U-test were used as appropriate. Any correlation between data was tested with the Spearman and Pearson correlation analysis. In addition, multivariate logistic-regression analysis that included potential confounders (age, glucose, creatinine, sex, ejection fraction, mean platelet volume, eosinophil and basophil) for CSFP was performed. Inclusion of variables into the final models was based on both clinical and statistical considerations. A probability value <0.05 was considered the minimum level of statistical significance. A two-sided p-value was considered for all comparisons.

## RESULTS

Seventy eight of 3368 patients had CSFP (Group I), and their demographic and laboratory findings were compared with 61 patients with normal coronary flow (Group II). Both groups were similar with respect to basic demographic characteristics including age, gender, dyslipidemia, hypertension, diabetes mellitus, smoking, serum glucose and left ventricular ejection fraction (Table-I). In Group I, coronary slow flow was observed in 174 coronary arteries. In 11 patients (14.1%), CSFP was found in a single artery, whereas in 67 patients (85.9%) multiple coronary artery involvement was observed. cTFC values of the CSFP group were significantly higher compared to those of the control group (38.9±12.5 frames versus 19.7±4.1 frames, p<0.001). 

Evaluation of blood parameters showed higher values of hemoglobin and hematocrit values, and eosinophil and basophil counts in the Group I (Table-II). Hemoglobin level was 14.2±1.4 g/dL in the CSFP group, and 13.6±1.7 g/dL in the control group (p=0.047). Hemotocrit level in the CSFP group was 41.9±3.8% versus 39.8±4.7% in the control group (p=0.005). Eosinophil ratio was 3.0±2.3% in the CSFP group, and 1.9±1.7% in the control group (p=0.004). Basophil ratio was 0.6±0.9% in the control group, whereas it was 0.9±1.1% in the CSFP group (p=0.045). Similarly, eosinophil and basophil counts were significantly increased in the CSFP group (p=0.001 and p=0.002, respectively).

**Fig.2:**
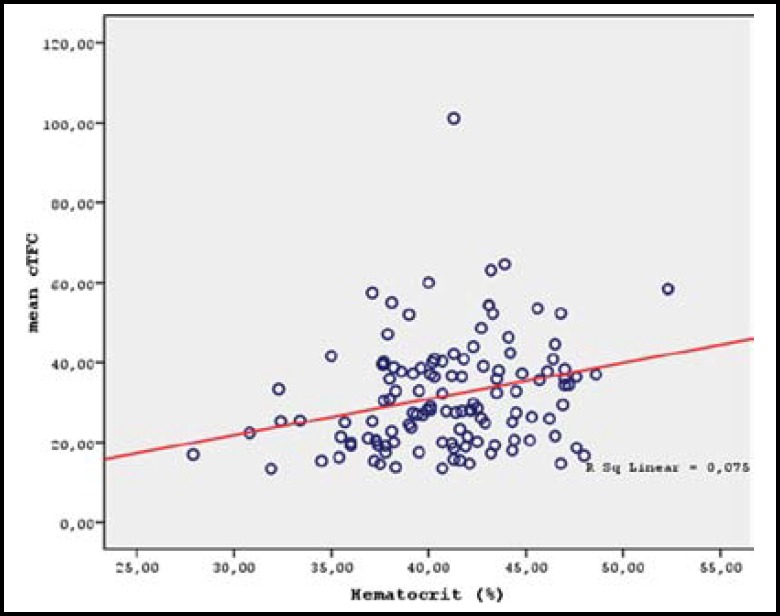
Correlation of mean TFC with hematocrit level (r=0.288 p=0.001).

**Fig.3:**
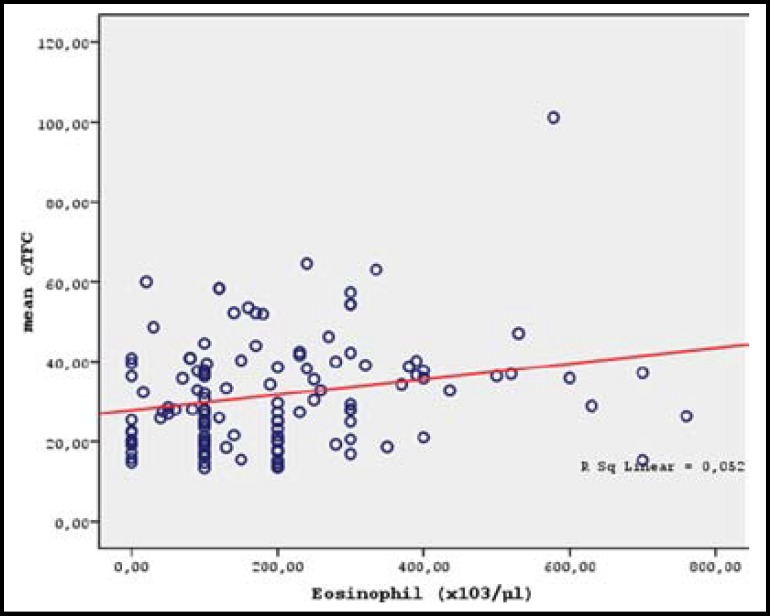
Correlation of mean TFC with Eosinophil count (r=0.217, p=0.014)

There were significant positive correlations between cTFC values and erythrocyte counts (r=0.230, p=0.009), hemoglobin levels (r=0.215, p=0.015), hematocrit values (r=0.288, p=0.001), and eosinophil counts (r=0.217, p=0.014) (Table-III and Fig. 2-3). In the multivariable regression analysis, only hematocrit value (OR=1.126, 95% CI: 1.030-1.231, p=0.009) and eosinophil count (OR=1.004, 95% CI: 1.001-1.006, p=0.006) were significant independent predictors of the presence of SCFP (Table-IV).

## DISCUSSION

The main anomaly in the pathophysiology of the CSFP is still under dispute. Some studies provide evidence in favour of a microvascular anomaly as the major cause.^7^^,^^8^ On the other hand, the rate of blood flow in the vascular bed does not depend entirely on vascular determinants. Blood viscosity, erythrocyte deformability and the intrinsic blood electrical resistance are also significant determinants of blood flow rate.^13^ Hematocrit value has been shown to be the most important determinant of blood viscosity in many studies.^14^^-^^16^ In addition to this, an in vivo study has demonstrated a positive correlation between the hematocrit value and the intrinsic blood electrical resistance.^17^ It has also been reported that a one unit rise in the hematocrit value contributes a 4% rise to blood viscosity.^15^

**Table-I:** Baseline characteristics of the study groups

	***Group I (n=78)***	***Group II (n=61)***	***p Value***
Age (years)	55.7±12.4	56.4±13.1	0.714
Men (%)	52 (66.7)	37 (60.7)	0.464
Hypertension (%)	38 (48.7)	31 (50.8)	0.865
Diabetes mellitus (%)	11 (14.1)	10 (16.3)	0.812
Smoke (%)	17 (21.7)	12 (19.6)	0.835
Low-density lipoprotein (mg/dl)	109.9±32.3	107.9±29.8	0.887
High-density lipoprotein (mg/dl)	43.1±11.0	45.6±12.8	0.260
Triglyceride (mg/dl)	150.1±79.0	143.6±100	0.146
Total cholesterol (mg/dl)	183.4±39.8	180.9±37.3	0.878
Serum glucose (mg/dl)	111.2±37.3	110.5±28.4	0.855
Ejection Fraction (%)	57.1±4.2	56.7±4.9	0.895
Serum Creatinin (mg/dl)	0.85±0.3	0.87±0.4	0.352
Symptoms at admission			
Chest pain (%)	53 (68)	44 (72)	0.594
Dyspnea (%)	19 (24)	16 (26)	0.800
Palpitation (%)	3 (4)	1 (1.6)	0.439
Syncope (%)	3 (4)	0	0.121
Corrected TIMI frame count			
Left anterior descending (frames)	43.38±22.62	19.56±4.88	**<0.001**
Circumflex coronary arter (frames)	32.60±16.21	17.43±4.57	**<0.001**
Right coronary artery (frames)	40.68±15.72	22.04±3.41	**<0.001**
Mean cTFC (frames)	38.9±12.5	19.7±4.1	**<0.001**
Slow flow at multiple coronary arteries (%)	67 (85.9)		
Slow flow at single coronary artery (%)	11 (14.1)		
Slow flow-related coronary artery	174		
Left anterior descending artery	70 (89.7)		
Circumflex coronary artery	40 (51.2)		
Right coronary artery	64 (82.0)		

**Table-II:** Hemorheological parameters of the study population

	***Group I (n=78)***	***Group II (n=61)***	***P Value***
Red blood cell (x10^3^/μl)	4.80±0.47	4.65±0.60	0.088
Hemoglobin (g/dl)	14.2±1.4	13.6±1.7	**0.047**
Hematocrit (%)	41.9±3.8	39.8±4.7	**0.005**
Red blood cell distribution width (%)	13.9±1.6	14.2±1.7	0.082
White blood cell (x10^3^/μl)	8.00±3.28	7.98±2.37	0.270
Neutrophil (%)	60.7±10.1	62.4±11.6	0.566
Neutrophil (x10^3^/μl)	5.02±2.25	5.20±3.07	0.514
Lymphocyte (%)	27.7±8.9	27.5±10.3	0.957
Lymphocyte (x10^3^/μl)	2.10±0.64	2.03±0.71	0.542
Monocyte (%)	6.7±2.7	7.0±2.1	0.087
Monocyte (x10^3^/μl)	0.58±0.77	0.54±0.28	0.811
Eosinophil (%)	3.0±2.3	1.9±1.7	**0.004**
Eosinophil (x10^3^/μl)	0.22±0.17	0.14±0.14	**0.001**
Basophil (%)	0.9±1.1	0.6±0.9	**0.045**
Basophil (x10^3^/μl)	0.056±0.06	0.036±0.07	**0.002**
Platelet (x10^3^/μl)	255.8±63.2	237.2±65.2	0.060
Mean platelet volume (fl)	8.1±1.2	8.4±1.1	0.251

**Table-III:** Correlations (Spearman) of mean cTFC with baseline clinical, biochemical and hemorheological parameters

***Variable***	***mean cTFC***	
	***r***	***P***
Age	-0.062	0.495
Sex (men)	-0.128	0.148
Left ventricular ejection fraction	-0.180	0.274
Serum glucose	0.041	0.661
Serum creatinine	0.013	0.886
Total cholesterol	0.119	0.233
Triglyceride	0.004	0.971
High-density lipoprotein	-0.077	0.438
Low-density lipoprotein	0.165	0.102
Red blood cell	0.230	**0.009**
Hemoglobin	0.215	**0.015**
Hematocrit	0.288	**0.001**
Red blood cell distribution width	0.037	0.680
White blood cell	0.026	0.768
Neutrophil	-0.018	0.842
Lymphocyte	0.115	0.197
Monocyte	0.031	0.726
Eosinophil	0.217	**0.014**
Basophil	0.162	0.068
Platelet	0.079	0.376
Mean platelet volume	0.061	0.493

**Table-IV:** Independent predictors of CSFP (n=139).

	***Univariate Logistic Regression***			***Multivarite Logistic Regression Analysis ***		
***Predict***	***Odds Ratio***	***95% CI***	***P Value***	***Odds Ratio***	***95% CI***	***P Value***
Sex (male)	0.820	0.408-1.645	0.576			
Serum glucose, mg/dl	0.863	0.991-1.011	1.001			
Age (years)	0.996	0.970-1.023	0.765			
LVEF (%)	1.012	0.971-1.054	0.581			
Serum Creatinin (mg/dl)	0.869	0.291-2.597	0.802			
Hematocrit (%)	1.130	1.037-1.232	0.005	1.126	1.030-1.231	0.009
Mean platelet volume (fl)	0.835	0.623-1.118	0.226			
Eosinophil (x10^3^/μl)	1.004	1.001-1.006	0.003	1.004	1.001-1.006	0.006
Basophil (x10^3^/μl)	1.005	0.999-1.011	0.111			

Abbreviations: LVEF, Left Ventricular Ejection fraction.

In our study we have shown a significant increase in the hematocrit values of the CSFP patients, which had a positive correlation with the cTFC values. Therefore, we can speculate that an increase in the erythrocyte concentration may cause slowing down of the coronary blood flow by increasing blood viscosity. Similarly, in a recent study, a positive correlation between hematocrit and cTFC values was shown in the absence of any significant coronary narrowing.^18^ Another study on CSFP patients without critical narrowing has also reported a significant increase in the hematocrit values.^19^ In contrast, in a limited number of studies using Cone/Plate Viscometer, rise in the blood viscosity could not be demonstrated in the CSFP patients.^19^^-^^21^ Conflicting results have been reported on the erythrocyte aggregation assessments. In vitro rotational viscometers were designed for relatively low hematocrit levels (maximum 40%) and for use at specific shear rates, which constitute a serious limitation for measurements obtained.^22^^,^^23^ For example, in studies where microvascular blood flow was simulated, Alizadehrad et al. have shown that at high hematocrit levels, changes in shear rate and arterial diameter caused a greater variation in the blood viscosity.^23^ Thus, the reported measurements with viscometers may not reflect the true viscosity at increased hematocrit levels.

Eosinophils exert their effects on target tissues as well as other leucocytes by degranulation.^24^ More recent observations present evidence that supports the argument for the involvement of eosinophils in the development of coronary artery disease.^25^^,^^26^ A relationship between elevated eosinophil counts and increase in cardiovascular events has been demonstrated.^27^^,^^28^ Aside from atherosclerotic narrowing, in a study performed on patients with variant angina, severity of vasospasm has been linked to the level of increase in the peripheral eosinophil counts.^29^ In another study, it was shown that levels of the eosinophil cationic protein were elevated in the coronary artery disease and acute coronary syndrome.^30^ In our study, peripheral eosinophil counts of the CSFP group were found to be significantly increased. At the same time, this increase had a negative correlation with the coronary blood flow rate. These results suggest that eosinophils might play a direct or indirect role in the etiology of CSFP.

Basophils, which constitute the lowest percentage of cells in the circulation possess important immunomodulatory properties. There are few studies on the effects of basophils on the coronary artery disease. Mochizuki et al. have shown a relationship between the peripheral basophil counts and atherosclerotic risk factors.^31^ Toyama et al. have reported that after statin treatment of patients with coronary artery disease the improvement observed in the arterial wall stiffness was correlated with the increase in basophil counts.^32^ However, some studies have reported that histamine released by the basophils is associated with atherosclerosis.^33^^,^^34^ Another evidence for the role of basophils in the development of CSFP is the Koinus syndrome. The clinical acute coronary syndrome brought about in this condition is believed to arise from the vasospasm caused by the degranulation of the mast cells which can cause plaque erosion and/or rupture.^35^ In our study, basophil count and ratio was significantly increased in the CSFP group of patients.

***Study limitation: ***The study population was selected according to coronary angiography reports, so the number of patients with CSFP does not reflect true ratio of CSFP in real life. Also the number of patients without any comorbid diseases was very small, and both groups had comorbidities with potential for atherosclerosis. 

## CONCLUSION

We found significant elevations in the hematocrit level, and erythrocyte, eosinophil and basophil counts in the CSFP patients compared to those with normal coronary blood flow. Additionally, positive correlations between these parameters and decreased coronary blood flow rates were demonstrated. Although the causative mechanisms are unclear, the results presented here are to the best of our knowledge perhaps the first in the literature to demonstrate a significant increase in the eosinophil and basophil counts in cases diagnosed with CSFP.

## Authors Contribution:

**KS, OG, HY and SY **conceived, designed the study and prepared the manuscript.

**OG and HY** supplied materials besides other contributions.

**KS, AIS, HY and SY **did statistical analysis and review.

**GA, SD and ÖY **was involved in literature search.

**KS, HY, GA and AIS **did data collection and manuscript writing.

**SD, ÖY, AIS and MS **did review and final approval of manuscript.

## Notes

***Financial support:*** None.
